# Oscillometric blood pressure measurements on smartphones using vibrometric force estimation

**DOI:** 10.1038/s41598-024-75025-9

**Published:** 2024-10-31

**Authors:** Colin Barry, Yinan Xuan, Ava Fascetti, Alison Moore, Edward Jay Wang

**Affiliations:** 1 Electrical and Computer Engineering, UC San Diego, La Jolla, CA USA; 2 Design Lab, UC San Diego, La Jolla, CA USA; 3 Geriatrics, Gerontology and Palliative Care, UC San Diego Health, La Jolla, CA USA

**Keywords:** Biomedical engineering, Medical research

## Abstract

This paper proposes a smartphone-based method for measuring Blood Pressure (BP) using the oscillometric method. For oscillometry, it is necessary to measure (1) the pressure applied to the artery and (2) the local blood volume change. This is accomplished by performing an oscillometric measurement at the finger’s digital artery, whereby a user presses down on the phone’s camera with steadily increasing force. The camera is used to capture the blood volume change using photoplethysmography. We devised a novel method for measuring the force applied of the finger without the use of specialized smartphone hardware with a technique called Vibrometric Force Estimation (VFE). The fundamental concept of VFE relies on a phenomenon where a vibrating object is dampened when an external force is applied on to it. This phenomenon can be recreated using the phone’s own vibration motor and measured using the phone’s Inertial Measurement Unit (IMU). A cross device reliability study with three smartphones of different manufacturers, shape, and prices results in similar force estimation performance across all smartphone models. In an *N* = 24 proof of concept study of the BP measurement, the smartphone technique achieves a mean absolute error of 9.21 mmHg and 7.77 mmHg of systolic and diastolic BP, respectively, compared to an FDA approved BP cuff. The vision for this technology is not necessarily to replace existing BP monitoring solutions, but rather to introduce a downloadable smartphone software application that could serve as a low-barrier hypertension screening measurement fit for widespread adoption.

**Supplementary Information:**

The online version contains supplementary material available at 10.1038/s41598-024-75025-9.

## Introduction

This paper proposes a novel oscillometric smartphone BP measurement method that requires no hardware modifications, no physical attachments, nor any need for individual calibration with cuff-based BP measurements. The proposed measurement can be enabled solely with software by downloading a smartphone application on nearly any smartphone. It is NOT an “optical” BP measurement relying on pulse transit time (PTT), pulse arrival time (PAT), pulse wave analysis (PWA), or any other technique estimating the relative change in BP. The application performs absolute BP measurements based on the oscillometric method similar to that employed by automated BP cuffs, in accordance with the recommendation of the American Medical Association (AMA) and the American Heart Association (AHA)^1^.

**Figure 1:**
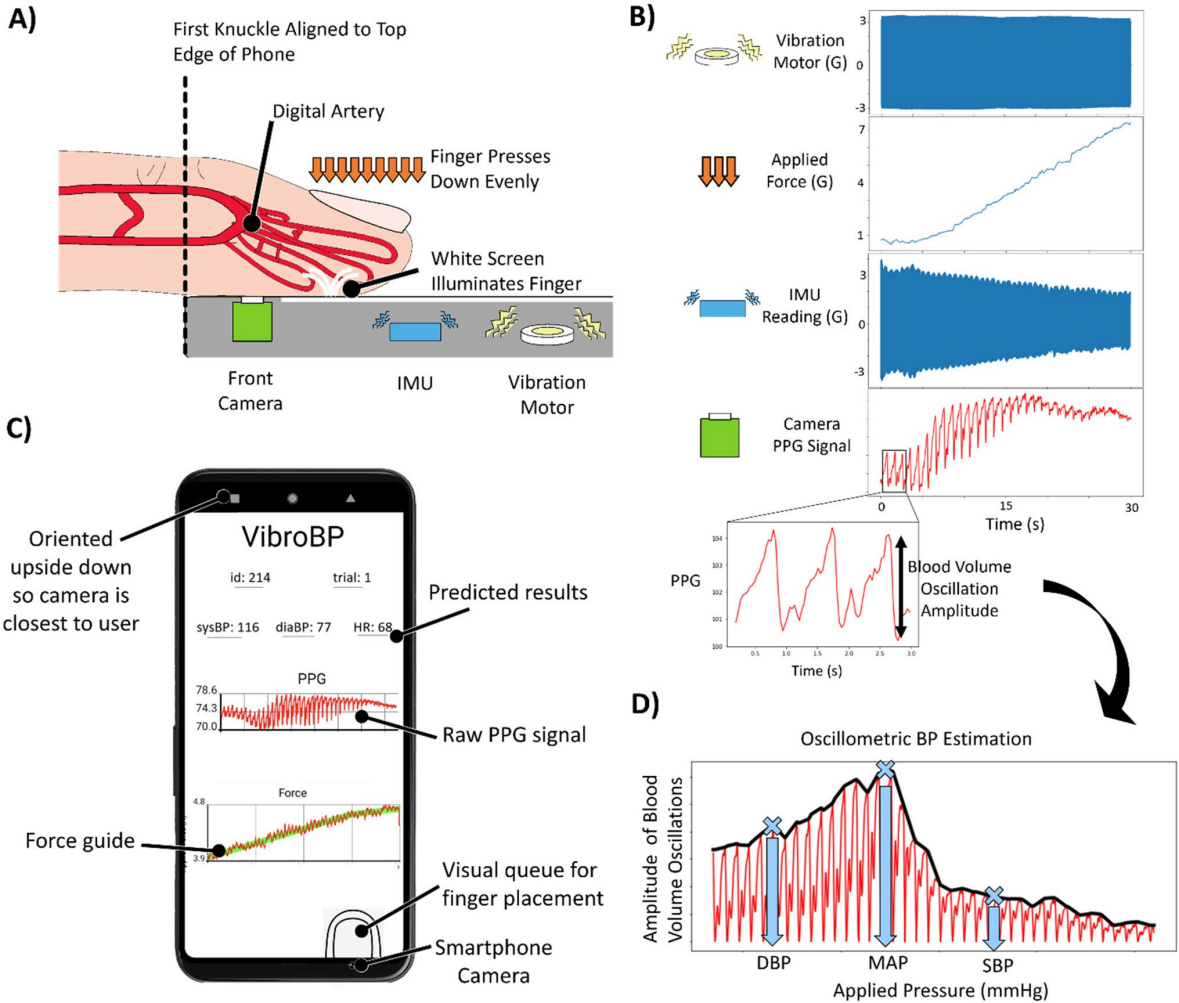
Project Overview. (**A**) Simplified diagram of the smartphone vibrometric force estimation, where the vibration motor drives oscillatory motion and the finger applies a force. The smartphone Inertial Measurement Unit (IMU) records the motion and the smartphone camera records PPG. (**B**) Applied finger force and linear y-axis acceleration of smartphone IMU are plotted vertically. As force increases, the oscillation amplitude of the y-axis acceleration decreases. The raw PPG signal is also plotted vertically to show how increased force affects the blood volume. (**C**) Smartphone app with visual queue for finger, guideline for applied force, and plot of PPG signal. (**D**) Amplitude of blood volume oscillations plotted against applied finger pressure. The approximate points of Diastolic Blood Pressure (DBP), Mean Arterial Pressure (MAP), and Systolic Blood Pressure (SBP) are indicated for interpretability.

The oscillometric method requires measuring two key metrics: applied force and local blood volume of an artery. Local blood volume of an artery can be estimated via photoplethysmography (PPG) with the smartphone camera. Measuring applied force with no attachments serves as the key insight presented in the paper to enable a novel BP measurement. Figure 1 depicts these key concepts of smartphone oscillometric BP measurements proposed in this paper.

To measure applied force, we propose a force estimation technique utilizing only the vibration motor and Inertial Measurement Unit (IMU). Nearly every phone contains an IMU with an accelerometer and gyroscope to measure the linear acceleration and angular velocity that enables phone position inferences used for rotating the screen, playing games, counting steps, gesture recognition, and much more. Similarly, nearly all smartphones contain a vibration motor to provide haptic feedback for messages, phone calls, and screen interactions. Taking advantage of the preexisting vibration motor and IMU allows us to create a model to measure force. The phone is set to maximally vibrate while the IMU measures the motion as the phone oscillates (from the vibration). When force is applied during vibration, the IMU signal changes in accordance with the altered oscillations. The alterations in the vibration signal allows us to train a model to estimate the applied force. We refer to this method of estimating applied force using the vibration motor and IMU signal as Vibrometric Force Estimation (VFE).

This work represents the first absolute BP measurement technique on a smartphone without special components or attachments. Most prior research into smartphone and smartwatch BP measurements perform relative BP measurements using PTT or PWA, but these techniques are fundamentally different. PTT and PWA only provide relative change in BP. These measures of relative change assume an individual has access to a BP cuff to calibrate their individual measurement due to the model’s reliance on arterial stiffness and arm length, otherwise, the relative measurements cannot be meaningfully interpreted^2–9^. Prior works on absolute smartphone BP measurement require custom built attachments to measure force^10,11^. One prior work functions only on a specific smartphone model with a built in force sensitive screen, a rare feature^12^. Our proposed method is the first smartphone BP measurement that requires no attachment and functions on nearly any smartphone model, only requiring a display screen, vibration motor, camera, and IMU.

## Results

### Blood pressure study participants

The full BP study recruitment included *N* = 30 participants, each with three resting smartphone BP measurements. From the 30 participants, 23 participants also participated in the exercise portion of the study for a total of 133 recorded smartphone BP samples. The participant population was 20% White, 67% Asian, and 13% Hispanic consisting of 50% males and 50% females. The mean age of the participants is 24.7 years with a standard deviation of 4.3 The average resting systolic and diastolic BP is 103mmHg and 70mmHg, respectively. Participant statistics are included in Table 1 with further details available in the supplementary materials. During the study, *N* = 29 participants were able to correctly apply force in at least 1 of their measurements as guided by the smartphone application. *N* = 24 participants contained at least 1 usable measurement (valid force and ppg signal combination) for BP prediction.

**Table 1:** Summary of participant data. This table summarizes participant BP measurement statistics. Note that resting and exercise BP are not separated or labeled in model training. Detailed information is available in the supplementary materials.

Measure	N	Min	Max	Mean	STD
Age (years)	24	18	38	24.7	4.3
Resting SBP (mmHg)	53	89	132	102.6	10.4
Resting DBP (mmHg)	53	60	84	69.8	6.6
Exercise SBP (mmHg)	15	107	145	129.5	109
Exercise DBP (mmHg)	15	78	107	88.8	8.9

The 133 samples consist of 90 normal samples and 43 exercise samples. Of the 133 samples, 65 were excluded based on the criteria detailed in the final paragraph of the methods section. For first time participants and participants performing exercise during the measurement, user error is more common so the high exclusion rate is expected. For the exercise samples, which involved participants performing a wall sit while taking a measurement, 28 of the 43 measurements are excluded. This resulted in usable exercise measurements from only 11 of the 23 exercise participants. For the non-exercise measurements, 37 of the 90 measurements are excluded. The data included in the BP model training and testing consisted of 53 non-exercise measurements and 15 exercise measurements. For detailed information on exclusion criteria for participant measurements, please refer to the last paragraph of the methods section.

### Blood pressure proof-of-concept study

**Figure 2:**
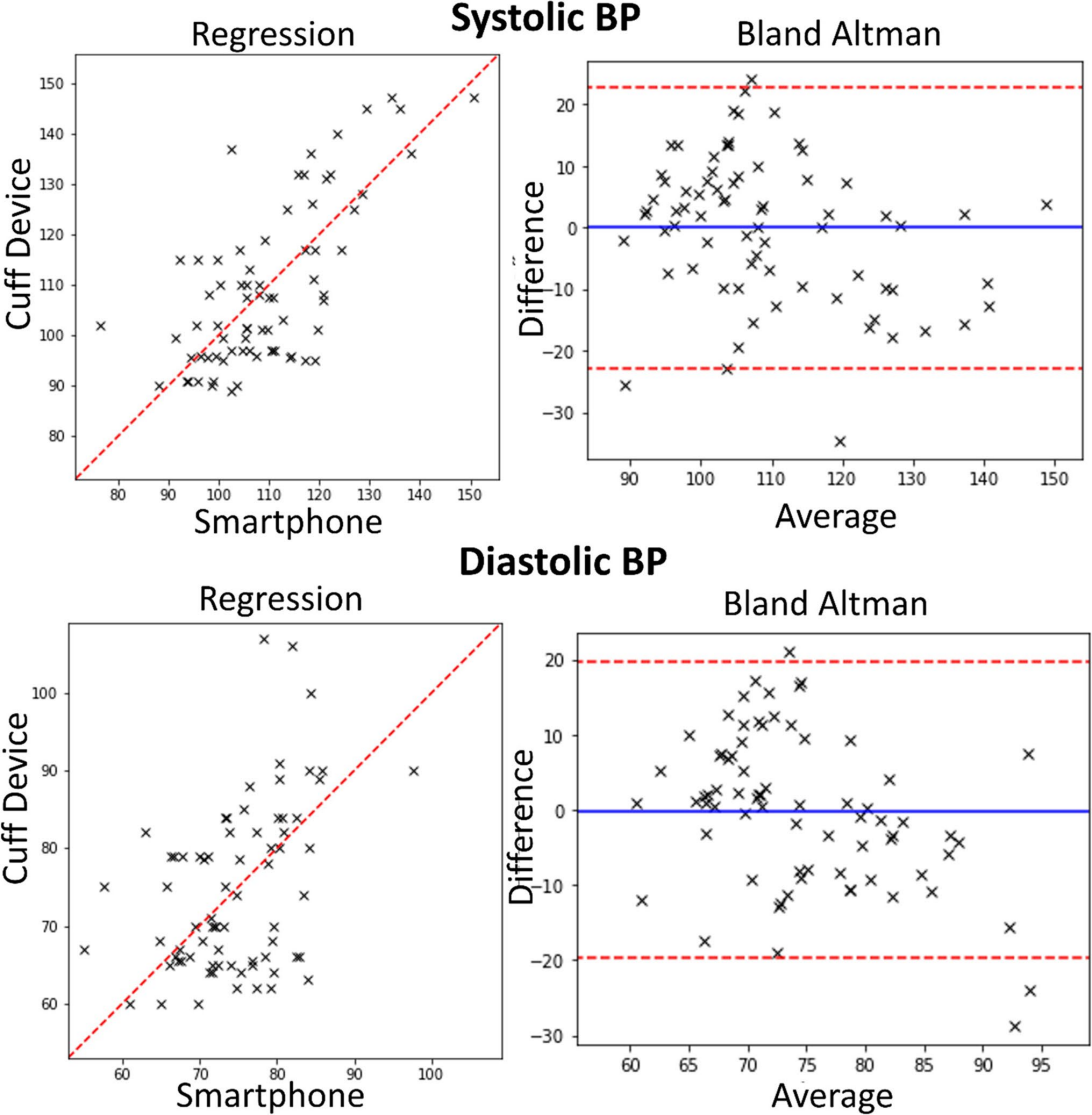
Blood pressure study results. Regression and Bland Altman plots for all measurements of *N* = 24 included participants. There are 68 total measurements because each participant can have multiple measurements.

The BP proof-of-concept study comparing the smartphone to the FDA-approved BP cuff with a hold one participant out validation achieves a mean absolute error of 9.21 mmHg and 7.77 mmHg for systolic and diastolic, respectively. For the same study, the mean and standard deviation of error is 0.09 ± 11.6 and − 0.24 ± 9.97 mmHg for Systolic and Diastolic, respectively. The Pearson correlation coefficient is 0.65 and 0.43 for systolic and diastolic measurements, respectively. The BP cuff measurements ranged from 89 to 147 mmHg systolic and 60 to 107 mmHg for diastolic. For the dataset, the mean and standard deviation is 108.6 ± 15 and 74.6 ± 10.7 mmHg for systolic and diastolic, respectively. These results are depicted in Fig. 2.

In evaluating the proof of concept more specifically around hypertension screening, we categorize cuff BP values based on the AMA/ACC standards^13^, which involves determining if an individual is below 120mmHg (normal), between 120 and 130 mmHg (elevated), and above 130 mmHg (hypertensive). In a binary categorizion of model results as normal/elevated (below 130mmHg systolic BP) or hypertensive (above 130mmHg systolic BP), the ROC analysis reveals an area under the curve (AUC) score of 0.92 for detecting high BP individuals, characterized by systolic BP greater than 130 mmHg. Under the same conditions, the model achieved a sensitivity and specificity of 0.86 and 0.89, respectively.

### Blood pressure group analysis

**Figure 3:**
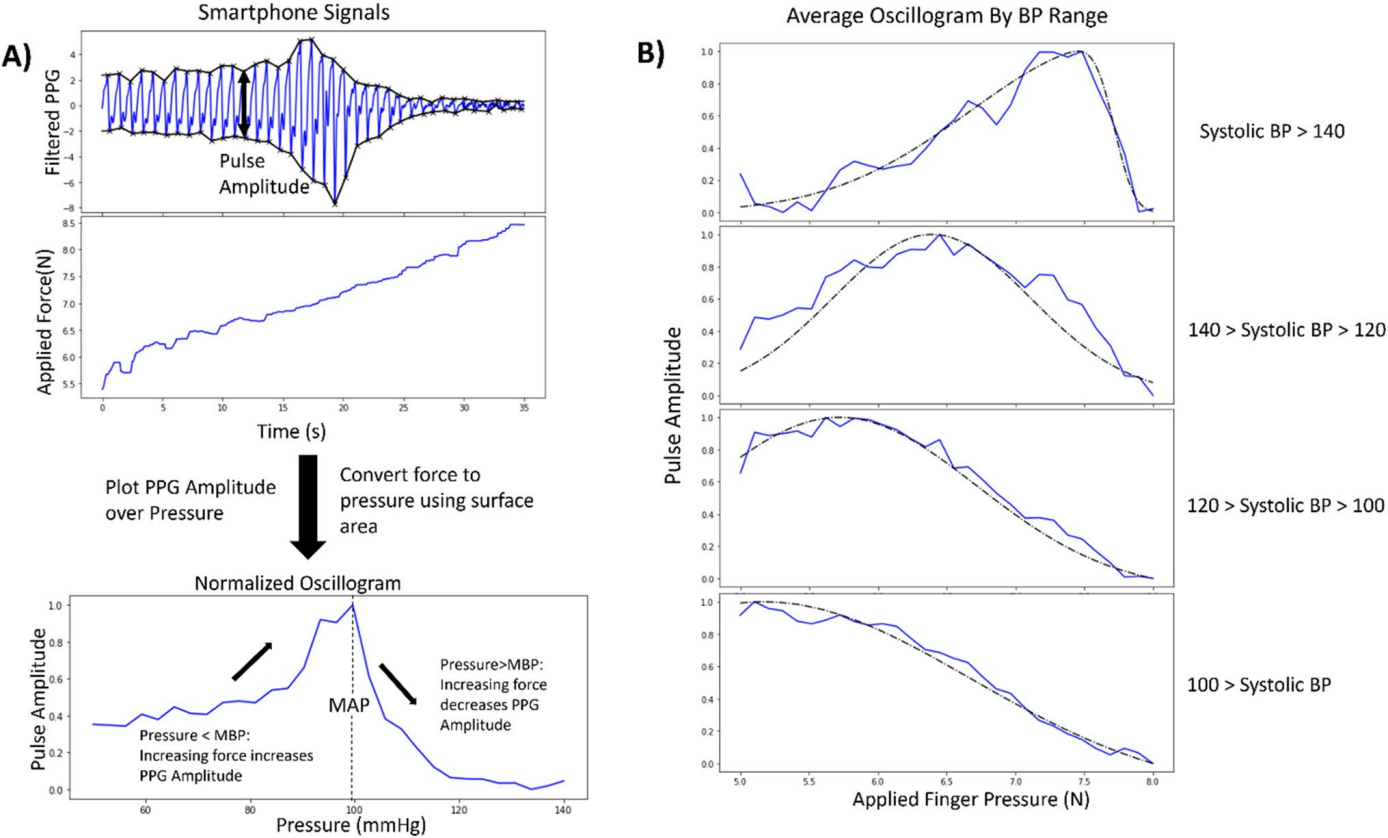
Oscillometric blood pressure measurements. (**A**) From the PPG and applied force signal captured on the smartphone, the amplitude of the filtered PPG signal is computed, and the force is converted to pressure via a fixed estimation of finger surface area. The filtered amplitude of the PPG signal plotted over the applied pressure is referred to as the oscillogram. The shape of the oscillogram resembles a skewed gaussian, where the peak approximates mean arterial pressure. (**B**) For each BP range, the average oscillogram is plotted to demonstrate the categorical differences. The average oscillogram shape is plotted as a solid line. The average skew gaussian fit is plotted as a dotted line. For higher blood pressure, the oscillogram peak shifts to the right because the mean arterial pressure is greater.

As shown in Fig. 3 part B, the oscillograms of each BP group contain characteristics supporting the clinical understanding of the oscillometric method. The most obvious characteristic is the peak of the oscillogram, often noted to represent the mean arterial pressure (MAP). As the MAP increases, the peak shifts right towards higher applied pressures.

### Skin tone and ethnicity

An ANOVA test is conducted to determine the impact of skin tone and ethnicity on performance. For both skin tone and ethnicity, the group variations are not statistically significant with p-values of 0.86 and 0.22, respectively. The participant population included 25% White, 17% Hispanic, 58% Asian participants with Fitzpatrick skin type ranging from type II to type V. The skin tone and ethnicity of each participant is listed in supplemental Table A.

## Intra-device validity

**Figure 4:**
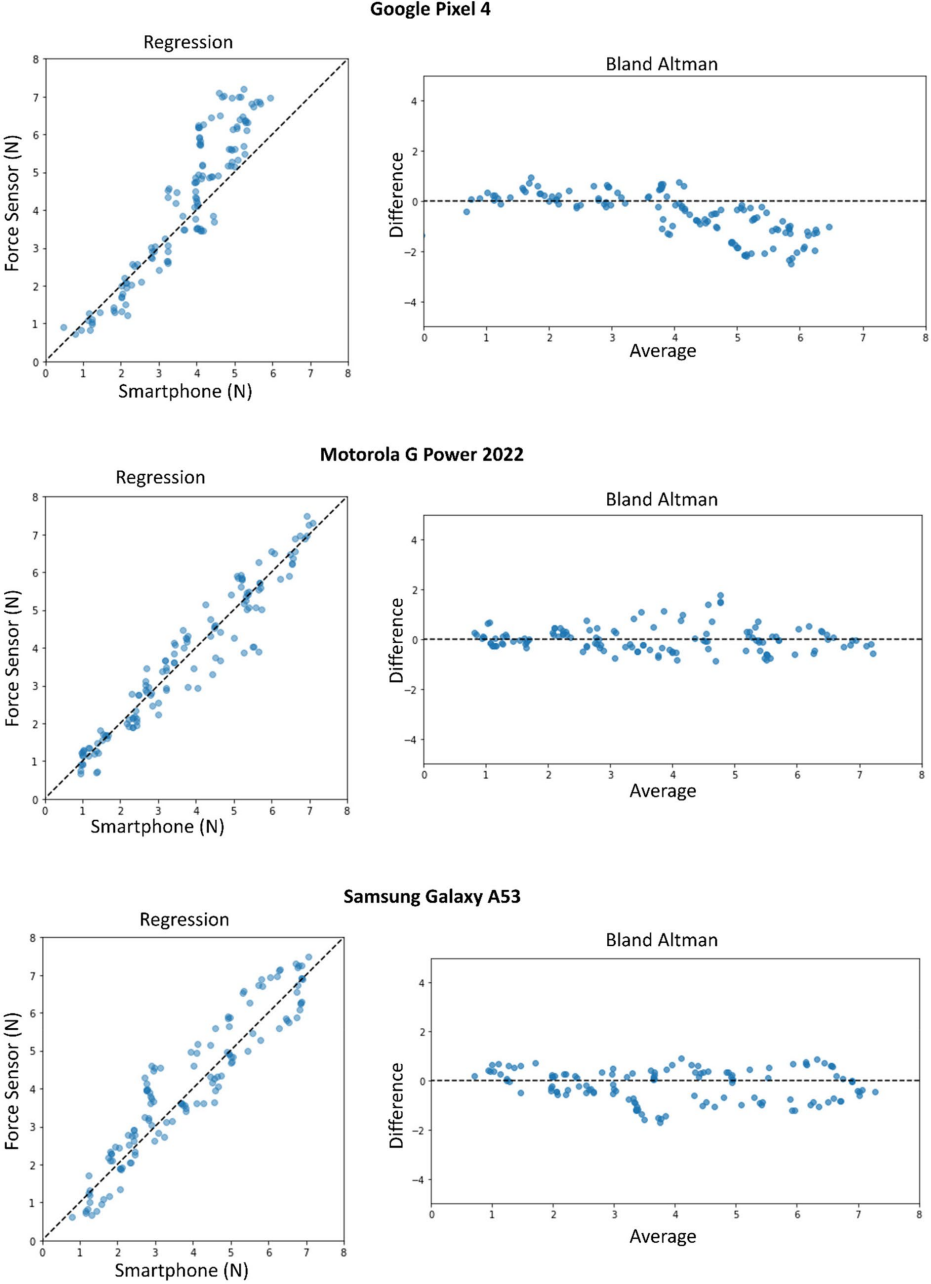
Force study results. Regression and Bland Altman plots of the force estimation performance of each smartphone compared to a linear, calibrated force sensitive resistor.

We ran a benchtop experiment demonstrating the force estimation technique across multiple models of smartphone. The accuracy of measuring applied finger force using VFE is evaluated across three smartphones: Google Pixel 4, Samsung Galaxy A53, and Motorola Moto G Power. The mean absolute error, standard deviation, bias, and correlation coefficient for the Google Pixel 4 is 0.77 N, 0.90 N, -0.46 N, and 0.92, respectively. The mean absolute error, standard deviation, bias, and correlation coefficient for the Samsung Galaxy A53 is 0.55 N, 0.64 N, -0.18 N, and 0.95 respectively. The mean absolute error, standard deviation, bias, and correlation coefficient for the Motorola Moto G Power 2022 is 0.39 N, 0.53 N, 0.01 N, and 0.96, respectively. The average correlation coefficient across all phones is 0.92. These results are visualized in Fig. 4.

### Intra-device validation

We trained a force estimation model on one Pixel 4 Smartphone then tested the force performance on four Pixel 4 smartphones: one new device, two refurbished devices, and one refurbished device with heavy use/wear. The MAE for each of the four devices is 0.97, 0.81, 0.71, and 1.45 N, respectively. Note that the model was trained on a refurbished Pixel 4. The device with heavy use performed significantly worse with the MAE of 1.45 N.

## Discussion

The study results demonstrate the feasibility of oscillometric smartphone BP measurements using VFE and PPG measurements. The systolic correlation coefficient of 0.65 and an MAE of 9.21 mmHg are comparable to prior smartphone-based BP measurements performed with attachments or custom equipment^10–12^. Additionally, the results suggest the smartphone measurement functions equally for groups of different ethnicities or skin tones. The central improvement offered in the work is the method for performing absolute BP measurements that can be applied across smartphone models without special features, modifications, or attachments.

Despite the promise, it is important to recognize the limitations of the proposed technology. The performance of the diastolic BP prediction is limited with a correlation coefficient of 0.42. This suggests that isolated diastolic hypertension could not be well detected; however, systolic BP alone can still accurately screen for hypertension in all but 3.2% of the population because the prevalence of isolated diastolic hypertension is extremely low compared to other types of hypertension^14^. Future exploration of data collection at lower force levels and features more specific to diastolic BP will likely improve the diastolic BP performance.

One key area for improvement is usability. During the blood pressure proof of concept study, the usability contributed to a significant proportion of the performance error and the large number of excluded measurements. For a valid measurement, a participant must apply force slowly, consistently, and with the correct technique. If the user performs poorly in applying the designated amount of force or if the user is constantly overcorrecting to apply force, it will likely cause BP measurement errors. Similarly, positioning the finger incorrectly or temporarily moving the finger off the camera during the measurement will result in error. Possibly the most critical usability challenge is ensuring that a user applies force through the entire finger rather than through the fingertip. Pressing with the fingertip may be most comfortable for some users accustomed to using the fingertip to interact with touchscreen interfaces; however, the fingertip is positioned above the smartphone camera so pulse amplitude changes from applied force at the fingertip alone are not detected.

The VFE technique is smartphone dependent. However, the calibration can be performed relatively quickly and once the profiling is completed for a phone model, it is likely VFE can work on all phones of that model. The intra-device performance suggests that even for devices with heavy use, the model does not break down entirely; rather, the variation in the vibration motor causes bias. It is expected that a baseline adjustment could account for this variation and this will be explored in future work. It would be expected that the application developer would perform the calibrations so the end user can simply download a ready-to-use application.

While the overall goal of this paper is to present and investigate the novel smartphone BP measurement methodology using vibrometric force estimation, there is significant potential for future work. The proof-of-concept studies performed here demonstrate feasibility, but future studies involving larger scale data collections of participants with varied hypertensive conditions will be performed to further develop vibrometric force estimation model parameters, train larger models, and better evaluate accuracy. Additionally, the studies performed thus far have all been performed under supervision in a controlled lab environment. There is a need for future work to explore the usability of the system for different demographics, especially focusing on the potential for individuals to perform measurements in at-home environments. Also, future research should include investigations of how conditions like diabetic neuropathy, Parkinson’s disease, or peripheral vascular disease might affect the measurement.

## Methods

### Blood pressure proof of concept study

In the BP proof of concept study, the smartphone device is compared against an FDA-approved cuff-based device (Omron BP7350). The participant first records a trial measurement using the smartphone to learn how to use the device and practice applying force. The trial measurement is not used for analysis. The cuff-based device is used to measure the participants BP following standard protocols. Immediately afterwards, the participant performs three BP measurements using the smartphone. After completing three BP measurements on the smartphone, the cuff-based device is used to measure the BP again. The full measurement sequence is as follows: cuff, smartphone, smartphone, smartphone, cuff. If the two cuff BP readings were successful, the two BP readings are averaged to provide a ground truth for all three smartphone measurements. If one of the cuff readings failed, the single successful cuff measurement serves as the ground truth.

The participants are also asked to optionally complete a second phase of the data collection involving exercise. The purpose of this exercise phase is to obtain high BP data. The *N* = 23 consenting participants are asked to perform a wall sit for approximately one minute. During the wall sit, the participants simultaneously measure their BP with the BP cuff device and the smartphone device using contralateral arms. Both BP measurements are initiated approximately ten seconds after the start of the wall sit.

The methods and experimental protocols described here are approved by the UC San Diego Internal Review Board (IRB) under protocol number 804668. The experiments were carried out in accordance with the relevant guidelines and regulations. Informed consent was obtained from all participants.

### Blood pressure study participants

A total of *N* = 30 participants were recruited for the BP proof of concept study. Participant demographic information is available in Table 1. Of the participants recruited, *N* = 6 participants were excluded from the data analysis. *N* = 1 participant was unable to perform any valid force measurements; *N* = 1 participant had low prominence in all measurements; and *N* = 1 participant had poor gaussian skew fit in all measurements. The other *N* = 3 excluded participants had a mixture of criteria below.

Participant measurements are excluded based on the following exclusion criteria:

- Cuff BP exceeds 155mmHg: If the reference measurement exceeded 160mmHg, the measurement is excluded based on prior research demonstrating that the fingertip and arm BP values differ at extremely high BP values induced from exercise^15^.
- Inconsistent/Undetected Pulse or Excessive Noise: The Fast Fourier Transform (FFT) of the PPG signal between zero and two Hz (frequencies relevant for human pulse) must have a narrow, identifiable peak indicating the pulse rate.
- Insufficient Applied Force: The applied force measurement must have a correlation coefficient greater than 0.88. The first 0.5 s of the force signal must have a correlation coefficient less than 0.21 N. Also, the minimum applied force must be less than six N and the maximum applied force value must be greater than seven N.
- Poor Skew Gaussian Fit: The mean absolute error for the normalized Skew Gaussian of best fit must be less than 0.18.
- Saturation in PPG signal: If the PPG signal rises above 254 pixel intensity units, the measurement is discarded. This is indicative of the finger being lifted off the smartphone camera during a measurement.
- Unchanging in PPG Prominence: The standard deviation of the normalized PPG prominence must be greater than 0.17.

### Smartphone application

For smartphone force estimation and BP studies, we develop a custom application called “VibroBP” to guide users and collect data. The smartphone application, shown in Fig. 1, provides the user with two key visual signifiers. First, the image of the fingertip near the smartphone camera provides an intuitive understanding of how to position the finger. Second, during the force measurement, the smartphone app plots the applied force in real time overlaid with a force guideline. The force guide aids the user to apply the correct amount of force during the measurement.

The application additionally provides the research staff with helpful information during data collection. The PPG signal is plotted in real time before and during the measurement so the research staff can ensure data quality. If the PPG signal is not within the desired range, the research staff can press a button to calibrate the PPG signal before the measurement to ensure the PPG signal is not over- or under- saturated. At the end of the study, the full force and PPG signals are plotted within the application to provide an overview of the data.

### Force dampening effect profiling

The proposed smartphone BP measurement method largely depends on accurately estimating force without an attachment. Each smartphone model has its own force dampening effect profile. To characterize the profile, a small-scale data collection is performed on each phone model to capture the vibration modulation at different known forces measured by a force sensitive resistor. This calibration procedure and all studies in the paper are performed on a flat wooden desktop surface.

We conducted this profiling for three different smartphones. With *N* = 5 participants of various hand sizes, a user applies pressure with their index finger onto a 0.3 mm thick force sensor positioned on top of the smartphone front camera. The user applies force with their index finger in the same vertical positioning as the BP measurement. The force sensor, a calibrated, linear force sensor (SingleTact 15 mm 4.5 N), is placed over the smartphone front camera. The user presses their finger on the force sensor (and smartphone camera) to measure the applied force of the finger and collect smartphone data.

During a measurement, real-time readings from the ground truth force sensor are displayed on a computer screen in front of the user with a force guide. The user is instructed to use real time feedback to follow the force guide in applying a range of pressures. Like the BP measurement, the force guide instructs users to continuously increase the applied force. The study includes three repetitions of a 40 s session of applying a range of forces. These three repetitions are performed by each participant on all three smartphones for a resulting 10 min of raw applied force data on each phone (2 min per participant).

### Vibrometric force estimation

For each smartphone, data from the force dampening effect profiling study provides IMU data during a range of applied force values. To estimate force, the IMU profile is used to train a machine learning model for each phone model. The IMU data contains three axis accelerometer data and three axis gyroscope data. Each axis of the IMU data contains high frequency signals from the vibration motor as well as noise at lower frequencies. The applied force from the finger most significantly affects the amplitude of the signal oscillation. To leverage this understanding, the rolling standard deviation of each axis of the accelerometer and gyroscope data serve as features. The features most significantly correlated to the force data are included as model inputs, while other features are excluded. With these IMU data features as inputs, a regression model (multivariate linear regression model or gradient boosting) is trained using a 10% holdout for validation. This process is repeated for each smartphone such that a distinct force estimation model exists for each smartphone model.

### Smartphone camera PPG

For the oscillometric BP measurement, we leverage the smartphone camera PPG to sense volumetric changes in blood flow at the transverse palmar arch branch of the digital artery. As the volume of blood flowing through the artery near the fingernail bed changes, the reflective properties of the tissue in the region changes. As such, the smartphone camera can record changes in blood volume by measuring the changes in the reflective properties.

In the study, the phone screen is set to a pure white background with maximal brightness and the user is instructed to place their index finger over the camera (as aided by a visual signifier). The bright white screen illuminates the finger and the camera records pixel intensity changes in the red channel as a proxy for blood volume changes. To account for skin tone and lighting variations, the smartphone performs the calibration outlined in Xuan and Barry et al.^16^ with the user’s finger over the camera. This calibration protects against under- or over- saturated PPG measurements.

### Oscillometry and blood pressure estimation

During the smartphone BP measurement, the smartphone camera records the PPG signal while the user exerts force with the index finger on the camera. Applying force with the index finger modulates the pulse amplitude in the PPG signal. A plot of pulse prominence through the range of applied forces creates a Gaussian or skew Gaussian-like shape that we refer to as the oscillogram. This oscillogram shape comes from the sigmoidal relationship between the transmural pressure and blood volume inherent from the elastic characteristics of arteries. As the applied pressure nears the internal pressure of the artery, the blood volume increases, resulting in an oscillogram peak near the MAP. 

The oscillometric method utilized by most FDA approved BP cuffs and numerous prior academic works utilize the oscillogram shape to estimate BP^7^. Our method of estimating BP relies on the features and findings identified in prior work.

We generate eleven features by focusing on features and concepts previously identified in prior work^17^ for oscillometric BP measurements. The following features are included in the BP estimation model: the applied force at the maximum prominence value, maximum gradient, minimum gradient, applied force at the maximum gradient, applied force at the minimum gradient, the maximum value of the oscillogram, prominence at the maximum gradient, prominence at the minimum gradient, applied force at the maximal low pass filtered PPG values, and applied force at the maximum gradient of low pass filtered PPG values. The concepts for these key features are visualized in Fig. 3 part A.

These key features of the oscillometric method are the only inputs into our BP prediction model. Note that the attributes related to exercise including heart rate and pulse shape are not included in the features provided to the model. Additionally, indirect measurements of heart rate or pulse are further avoided by interpolating all oscillograms to have the same number of points with the same force values. With the key features, we trained a least absolute shrinkage and selection operator (LASSO) regression model to predict systolic and diastolic measures of BP^18^. The model is trained and tested using a hold one participant out validation. In this training scheme, the model is trained on all data excluding the one or more BP measurements from a single participant. The model is then used to predict the BP of the holdout participant. This process is repeated for each participant.

For the hypertensive screening analysis, the smartphone predictions are categorically compared to the cuff predictions. The smartphone BP predictions are utilized with a universally added bias term for classification based on AMA/ACC standards^13^.

## Supplementary Information

Supplementary Material 1.

Supplementary Material 2.

Supplementary Material 3.

## Data Availability

Additional information, data, code for analysis, or materials relevant to the research are available upon request to the corresponding author, Colin Barry, at c1barry@ucsd.edu.

## Declarations

Colin Barry and Edward Jay Wang are inventors on a provisional patent pertaining to this manuscript with the attorney docket number 09062 − 8487 US00.
