# Supplementary material for: Oscillometric blood pressure measurements on smartphones using vibrometric force estimation
Source: Sci Rep. 2024 Oct 31;14:26206. doi: 10.1038/s41598-024-75025-9 (PMC11527996; doi:10.1038/s41598-024-75025-9)
Supplement: Supplementary file 3 — Supplementary Material 3. [file 41598_2024_75025_MOESM3_ESM.pdf]

Supplementary Materials

ID	Gender	Ethnicity	Age	Skin	Est. BP (mmHg)	Ref. BP (mmHg)	Ex. Est. BP (mmHg)	Ex. Ref. BP (mmHg)	#R	#E
0	Male	White	25	II	118 / 73	117 / 70	139 / 86	151 / 92	3 / 3	1 / 3
1	Male	White	26	IV	106 / 76	97 / 62	126 / 83	127 / 86	3 / 3	2 / 3
2	Male	Asian	24	V	110 / 83	101 / 66	--	118 / 75	2 / 3	0 / 3
3	Female	Hispanic	28	III	95 / 67	115 / 79	122 / 81	131 / 89	3 / 3	1 / 1
4	Male	Asian	21	III	97 / 66	91 / 65	--	--	1 / 3	0 / 0
5	Female	Asian	23	II	107 / 82	89 / 68	--	--	1 / 3	0 / 0
6	Male	Asian	23	III	104 / 71	117 / 68	--	167 / 113	1 / 3	0 / 1
7	Female	Asian	26	III	96 / 67	95 / 65	--	104 / 73	2 / 3	0 / 3
8	Male	White	25	II	112 / 71	103 / 64	--	147 / 85	1 / 3	0 / 1
9	Female	Asian	23	III	95 / 64	91 / 60	110 / 74	112 / 82	3 / 3	2 / 3
10	Female	White	21	III	91 / 66	102 / 67	--	136 / 90	3 / 3	0 / 1
11	Male	Asian	20	IV	105 / 70	97 / 66	--	--	3 / 3	0 / 0
12	Female	White	27	IV	101 / 66	96 / 68	142 / 90	140 / 96	1 / 3	1 / 2
13	Male	Asian	23	III	118 / 77	111 / 65	--	161 / 106	1 / 3	0 / 2
14	Male	Asian	21	V	112 / 76	95 / 64	--	115 / 89	3 / 3	0 / 1
15	Male	Hispanic	38	III	103 / 71	101 / 78	107 / 64	119 / 79	2 / 3	1 / 1
16	Male	Hispanic	28	II	118 / 79	132 / 74	--	152 / 99	2 / 3	0 / 3
17	Female	Asian	29	III	108 / 81	108 / 80	118 / 85	132 / 100	3 / 3	1 / 1
18	Male	White	25	III	104 / 73	110 / 65	125 / 79	143 / 89	3 / 3	2 / 3
19	Female	Asian	21	II	110 / 66	107 / 75	101 / 77	137 / 107	3 / 3	1 / 1
20	Female	Asian	22	II	97 / 72	90 / 70	119 / 85	125 / 106	3 / 3	1 / 1
21	Female	Asian	32	III	97 / 70	99 / 82	--	134 / 112	3 / 3	0 / 1
22	Female	Asian	18	III	105 / 75	96 / 84	--	122 / 105	3 / 3	0 / 1
24	Female	Hispanic	26	III	--	117 / 75	127 / 83	132 / 87	0 / 3	2 / 3

Supplemental Table A. Proof of Concept Study Participant Data. This table provides in depth participant BP measurement statistics. Ref. refers to the reference cuff measurement and Est. refers to the estimated measurements from the smartphone. #R and #E refers to the number of resting and exercise measurements included in the study. Some measurements are excluded due to usability or other errors as explained in the Methods section.

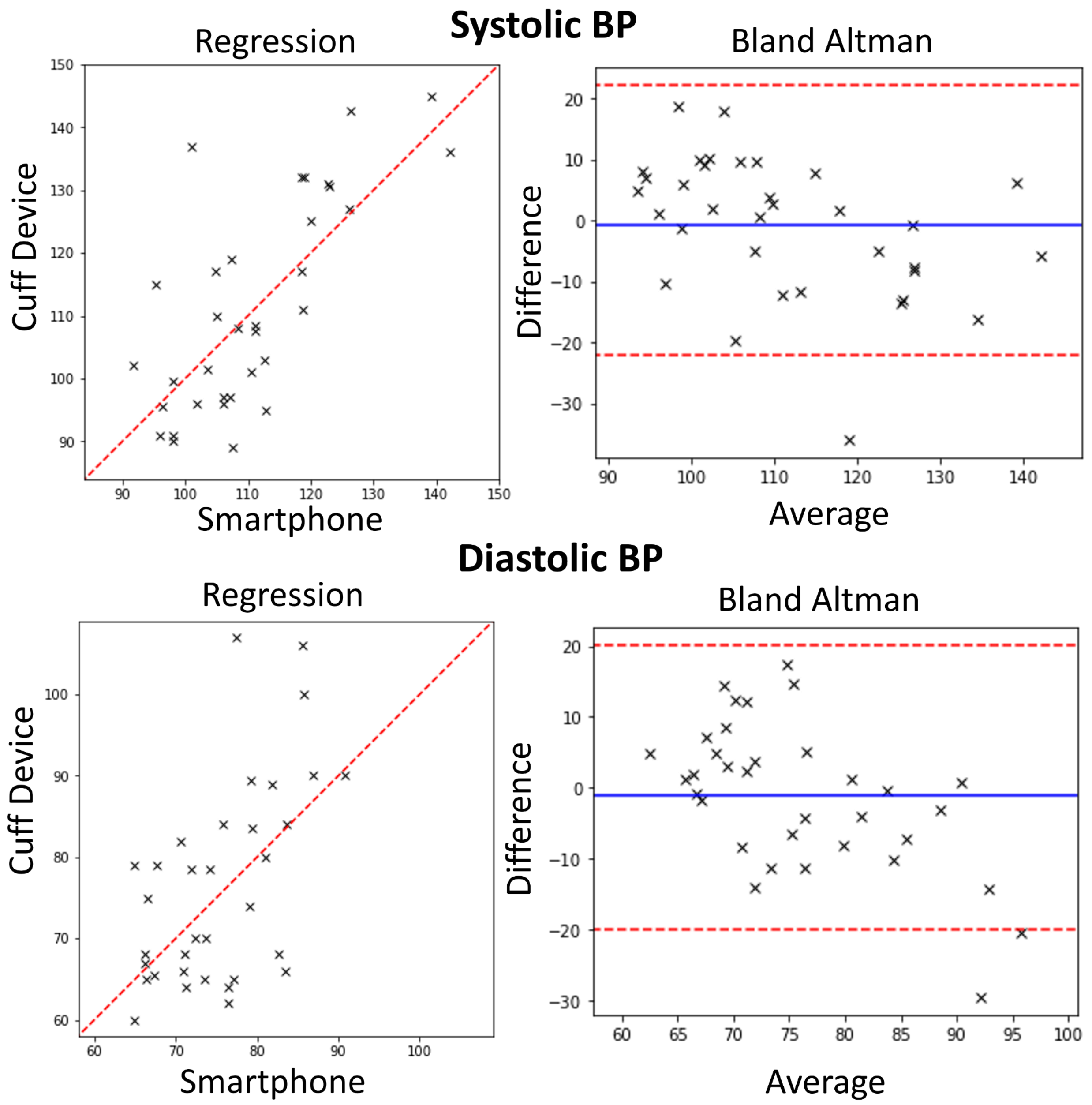

Supplemental Figure B. Blood Pressure Study with Participant Averages

This figure replicates Figure 2 with averaged participant data. Data from each participant is split into exercise and resting then averaged and plotted. Subsequently, this method of analysis results in a mean absolute error of 8.9 mmHg and 8.0 mmHg for systolic and diastolic, respectively. The mean and standard deviation of error is -0.9 ± 11.2 and -1.1 ± 10.2 mmHg for Systolic and Diastolic, respectively. The Pearson correlation coefficient is 0.73 and 0.56 for systolic and diastolic measurements, respectively.

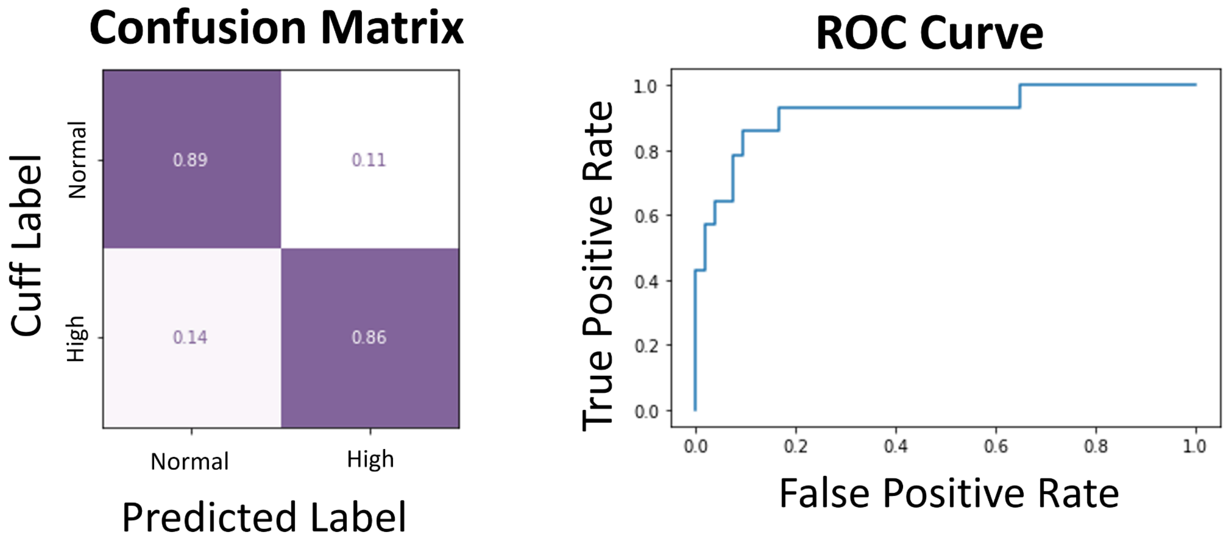

Supplemental Figure C. Classification Confusion Matrix and ROC Curve
These plots illustrate the potential performance of the proposed system as a hypertensive classifier based on the data from the blood pressure proof of concept study.

Publication	Sensors/ Components	SBP Bias Error	SBP Precision Error	SBP MAE	DBP Bias Error	DBP Precision Error	DBP MAE	SBP range	DBP range	Number of Participants
This paper	Smartphone vibration motor and IMU	0.09	11.6	9.21	-0.24	9.7	7.77	58	47	24
Xuan et. al. 2023	3D printed attachment with spring and pinhole	1.72	n/a	8.72	0.3	n/a	5.49	76	40	24
Chandrasekhar et. al. 2018-2	iPhone X with integrated pressure sensor	-4	11.4	n/a	-9.4	9.7	n/a	"40 to 50"	"40 to 50"	18
Chandrasekhar et. al. 2018-1	Electronic smartphone attachment with ppg and force sensors	3.3	8.8	n/a	-5.6	7.7	n/a	"about 50"	"about 50"	22

Supplemental Table B. Comparison to Prior Work This table provides a comparison of the proposed system to relevant previous work.

a) b)

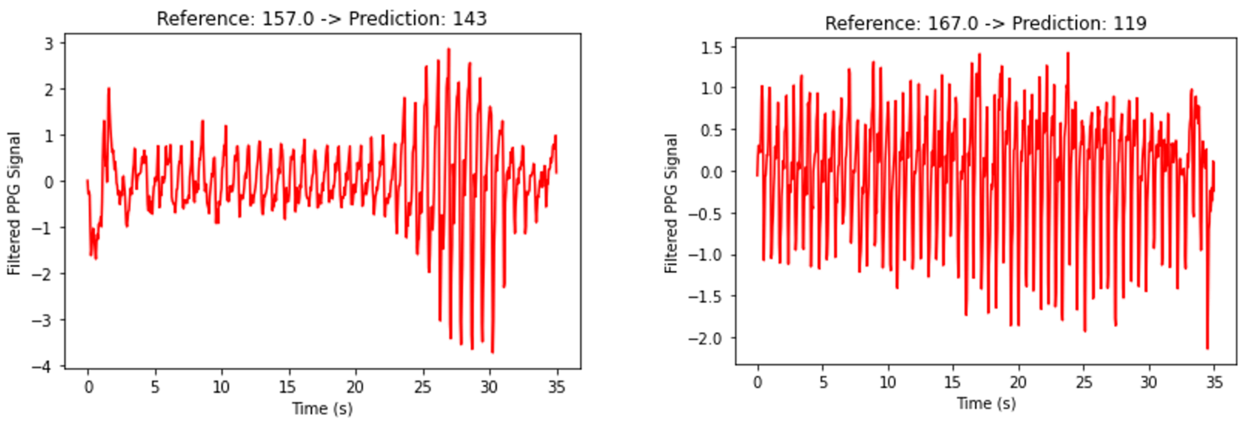

Figure D: Excluded high blood pressure measurements. The two measurements depicted above are the only measurements excluded based on their BP values. Both measurements contain force measurements with approximately linearly increasing applied pressure (not shown). The measurement from Part a) resembles the expected signal where the peak of the oscillogram is shifted towards the right (high blood pressure). The prediction is near the expected error and may be minimally affected by differences between upper arm and finger blood pressure from exercise induced high blood pressure. The measurement from Part b) resembles the concern about high blood pressure from this system; no definitive peak is present in the oscillogram. This is likely because either 1. There is poor measurement technique from the participant or 2. The set range of pressure that the participant applied is insufficient for this level of high blood pressure range.
